# Immobilization of Trypsin from Porcine Pancreas onto Chitosan Nonwoven by Covalent Bonding

**DOI:** 10.3390/polym11091462

**Published:** 2019-09-06

**Authors:** Jung Soo Kim, Sohee Lee

**Affiliations:** 1 Department of Biosystems & Biomaterials Science and Engineering, Seoul National University, Seoul 151-921, Korea; 2 Department of Clothing & Textiles, Gyeongsang National University, Jinju 52828, Korea; 3 The Research Institute of Natural Science, Gyeongsang National University, Jinju 52828, Korea

**Keywords:** enzyme, immobilization, chitosan, trypsin, nonwoven

## Abstract

The present study deals with the potential application of chitosan nonwoven for biomedical textiles based on enzyme immobilization. For this, chitosan nonwoven was first cross-linked with glutaraldehyde to introduce aldehyde groups at optimal conditions. To immobilize the enzyme trypsin onto glutaraldehyde-pre-activated chitosan nonwoven, several parameters such as pH, enzyme concentration, and reaction times were investigated. In addition, the pH, thermal stability, storage stability, and reusability of immobilized trypsin were examined. We found that the optimal immobilization conditions for trypsin were pH 8.5, enzyme concentration of 8% (owf), and treatment time of 30 min. Trypsin was immobilized at 25 °C efficiently. The immobilized trypsin showed lower pH stability and better thermal stability than free trypsin. The immobilized trypsin showed 50% of its initial activity after being used 15 times and 80% of that after 20 days of storage at 4 °C. SEM analysis also confirmed that trypsin was immobilized on chitosan nonwoven.

## 1. Introduction

Enzymes have superior catalytic activity under mild processing conditions, which leads to higher reaction yields and few side reactions. Due to their specific activities, enzymes have been studied and applied in various fields [1,2]. However, enzymes have limited applications in industrial processes because they cannot be easily reused and recycled and have low activities under non-optimal conditions and low stability [2,3]. To overcome these limitations, enzyme immobilization has been applied in the industry. Enzyme immobilization is a method to enhance enzyme convenience, stability, and reusability and is carried out by adsorbing, cross-linking, or entrapping an enzyme to a carrier. Each enzyme immobilization method has limitations such as enzyme denaturation, desorption/leaching, reduced activity, and process complexity unless optimized [4]. Nevertheless, enzyme immobilization is a convenient and economical method because it may increase enzyme stability and allow enzyme recovery and reuse [1,2]. In the textile industry, enzyme technology is drawing attention as an alternative to the traditional chemical finishing due to its characteristics of being non-toxic and environment-friendly [5,6,7,8,9]. In the fields of fiber modification, bio-bleaching, dye discoloration and decomposition, industrial wastewater treatment, and laundry detergent decontamination, various enzymes such as lysozyme, laccase, cellulase, amylase, and peroxidase have been studied for their possible immobilization [10,11,12,13,14,15,16,17].

**Figure 1:**
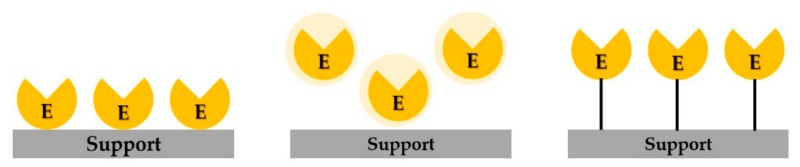
Enzyme immobilization techniques: (**left**) physical adsorption, (**middle**) entrapment (**right**) covalent bonding. E: enzyme.

The method of enzyme immobilization comprises physical absorption, entrapment, and covalent bonding (Figure 1). Entrapment consists in immobilizing an enzyme by inserting it into the fine grid of a gel or polymer membrane that does not interact chemically with the enzyme; this increases the enzyme stability by limiting its folding or modifications within the polymer lattice network. However, the limited space in which the enzyme is confined by entrapment could affect its activity [3,4,18]. By physical adsorption, an enzyme is adsorbed to and immobilized on a carrier; however, due to the weak bonding between the enzyme and the carrier, the adsorbed enzyme can be easily detached by external factors, such as temperature, pH, and salt concentration [3,18]. Immobilization through covalent bonds is the most widely used method to immobilize an enzyme by establishing covalent bonds between amino acid residues of the enzyme that are not involved in its activity and radicals on the surface of the carrier [3,18]. Due to the strong bonds between the carrier and the enzyme, immobilization by covalent bonding has advantages: the enzyme is not separated easily during a reaction, limiting the enzyme leakage from the support, and the thermal stability of the enzyme is increased [3,10,18]. However, covalent immobilization has limitations: high risk of enzyme denaturation and, sometimes, decreased enzyme activity in affinity reactions [18].

A suitable carrier for immobilization is important because the carrier affects enzyme activity, stability, and reactivity during immobilization [1]. In order to be used as an immobilization carrier, proper mechanical strength, sufficient reactive functional groups, high protein affinity, physical and chemical stability, affordability, hygienic innocuousness, and ease of molding are required [3,19,20]. Chitosan is a well-known carrier for immobilization because of its physical safety, antimicrobial, deodorizing, adsorption, and humectant properties, and economic convenience [21,22]. In addition, its excellent biocompatibility and biodegradability make it an attractive compound in a variety of fields. Moreover, chitosan has a multifunctional group and therefore it can easily combine with glutaraldehyde which can be used as a cross-linked reagent [22]. Thus, many studies have been conducted on enzyme immobilization with chitosan as a carrier [22,23,24,25,26]. In a nonwoven form, it has the advantages of high specific surface area, high mechanical strength, chemical stability, low diffusion resistance of substrates and products, as well as durability when used for enzyme immobilization [22]. In addition, it is attracting attention in the textile industry due to its light weight, ease of handling, and affordability [24,25,26,27]. Although electrospinning of pure chitosan is still limited, chitosan-based electrospun fibrous mats, a type of nano-to-micron-sized nonwovens, have the advantages of high surface-to-volume ratio and tailored porosity enabling excellent mass transfer [28,29]. As-spun chitosan–(Poly(ethylene oxide) (PEO) mats were immobilized with glucose oxidase (GOX) to implement an in-situ hydrogen peroxide (H_2_O_2_) generation system [28]. Chitosan nonwoven is being studied as a carrier due to its high biocompatibility, excellent breathability, absorbency, easy adherence to the skin due to its flexibility, and no antigenicity [22,27].

Among suitable enzymes for immobilization, trypsin is widely used in the medical, pharmaceutical, food, and biology fields and it has the highest substrate specificity among proteases [30,31,32,33]. Trypsin is used in the textile industry as a functional enzyme, being added to laundry detergents in order to increase the removal of protein soils [34], and is used in medicine as an anti-inflammatory enzyme in the treatment of superficial or internal inflammations [30]. Studies are being conducted on the wound-healing effect of immobilized trypsin, as well as on its use as a functional material. Monteiro et al. (2007) evaluated the functionality of immobilized trypsin on cellulose gauze as a wound dresser [35]; Nikolic et al. (2010) reported on the storability of trypsin immobilized using cotton threads, applicable as a bandage [31]. When using trypsin as an immobilization enzyme, setting optimal immobilization conditions is required because the external conditions can reduce the efficiency and the activity of the enzyme.

Therefore, the aim of this present study was to investigate trypsin immobilization onto glutaraldehyde-activated chitosan nonwoven. For this, the immobilization conditions for trypsin onto glutaraldehyde–chitosan nonwoven were determined by evaluating various parameters such as pH, enzyme concentration, and reaction time. Second, the pH stability and thermal stability of free and immobilized trypsin were compared. Third, the storage stability and reusability of immobilized trypsin were evaluated. Fourth, trypsin immobilization on chitosan nonwoven was confirmed through the observation of surface morphology. Based on the present study, optimal trypsin immobilization conditions with glutaraldehyde-activated chitosan nonwoven for the development of potential functional textiles were established.

## 2. Materials and Methods 

### 2.1. Materials

The characteristics of chitosan nonwoven and trypsin used in this study are described in Table 1 and Table 2. 

The buffer solution to maintain a constant pH during pre-activation of chitosan nonwoven with glutaraldehyde and enzyme immobilization was composed of: Trizma base (Sigma Chemicals Co., St. Louis, MO, USA), Trizma HCl (Sigma Chemicals Co., St. Louis, MO, USA), sodium phosphate monobasic (Sigma Chemicals Co., St. Louis, MO, USA), sodium phosphate dibasic (Sigma Chemicals Co., St. Louis, MO, USA), sodium carbonate (Duksan Pure Chemicals Co., Seoul, Korea), and sodium bicarbonate (Duksan Pure Chemicals Co., Seoul, Korea). Glutaraldehyde (Junsei Chemical Co., Japan) was used as a cross-linking agent at a concentration of 25%in H_2_O. Nα-Benzoyl-L-arginine 4-nitroanilide hydrochloride (L-BAPA, Sigma Chemicals Co., St. Louis, St. Louis, MO, USA), dimethyl sulfoxide (DMSO, Sigma Chemicals Co., St. Louis, St. Louis, MO, USA) and calcium chloride (Kanto Chemical Co., Tokyo, Japan) were used for the measurement of trypsin activity. All chemicals were used without further purification. 

### 2.2. Experimental Method

#### 2.2.1. Preparation of Pre-Activated Chitosan Nonwoven with Glutaraldehyde

Chitosan nonwoven was pre-activated with glutaraldehyde at the optimal treatment conditions according to previous work [36]. A previous study indicated glutaraldehyde pretreatment conditions concerning pH, glutaraldehyde concentration, and crosslinking time for chitosan nonwovens to increase the activity of immobilized enzymes. The procedure applied was: chitosan nonwoven (0.15 g) was added to a 25 mM sodium carbonate buffer (pH 10.0) at a ratio of 1:50 in the presence of glutaraldehyde 3% (v/v), and the mixture was stirred for 2 h at 25 °C. Excess glutaraldehyde on chitosan nonwoven was removed by washing several times with distilled water.

#### 2.2.2. Immobilization of Trypsin onto Glutaraldehyde-Pre-Activated Chitosan Nonwoven

**Figure 2:**
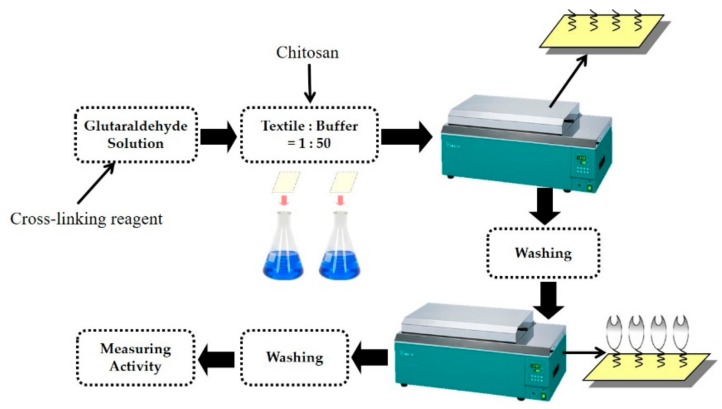
Schematic presentation of the trypsin immobilization process onto chitosan nonwoven.

Trypsin immobilization on glutaraldehyde-pre-activated chitosan nonwoven was carried out in different immobilization conditions in a 25 mM buffer solution at a solution ratio of 1:50. Trypsin immobilization was evaluated depending on the pH (7.0–10.0), trypsin concentration (2–14%, on-weight-fabric (owf)), and reaction time (0.25–4 h) at 25 °C and 110 rpm in a shaking water bath (BS-31, JEIO TECH Co., Seoul, Korea). Trypsin–chitosan nonwoven was washed five times until no residual trypsin was detected in distilled water, and the activity of immobilized trypsin was measured after one-hour storage at 4 °C (Figure 2) [31].

#### 2.2.3. Immobilized-Trypsin Activity Assay

Immobilized-trypsin activity was measured with L-BAPA. Chitosan nonwoven fabric with immobilized trypsin under reaction conditions was immersed in 10 mL of 50 mM Tris buffer that contained 10 mM calcium chloride [32], and 35 μL of 50 mM L-BAPA dissolved in DMSO was then added. The reaction was carried out in a shaking water bath at 110 rpm and 25 °C for 10 min. The absorbance of the supernatant from the reaction was measured using a UV–Vis spectrophotometer (M-3100, SCINCO CO., Seoul, Korea) at 410 nm [26]. The optimal conditions were determined by calculating the relative enzyme activity using the following expression:

Relative activity (%) = A_1_/A_0_ × 100

where A_0_ is the maximum absorbance of the soluble or immobilized trypsin in reaction conditions, and A_1_ is the absorbance of the soluble or immobilized trypsin in different conditions.

#### 2.2.4. pH and Thermal Stability of Free and Immobilized Enzyme

The pH and thermal stability of free and immobilized trypsin were measured in the pH range of 6.0–11.0 at 25 °C and in the temperature range of 25–65 °C at a pH of 8.5. In this experiment, 0.5 mg/mL of free trypsin and trypsin immobilized on chitosan nonwoven were stored at different conditions for 1 h without substrate; then, 35 μL of 50 mM L-BAPA dissolved in DMSO was added, and the reaction was carried out for 10 min at 25 °C. Trypsin activity was measured as described above. The pH and thermal stability of free and immobilized trypsin were compared and evaluated by calculating the relative activity (%).

#### 2.2.5. Storage Stability of Immobilized Trypsin

The storage stability of immobilized trypsin was evaluated by measuring the activity of immobilized trypsin after storing the immobilized-trypsin–chitosan nonwoven for 20 days at 4 °C [1]. 

#### 2.2.6. Reusability of Immobilized Trypsin

The reusability of immobilized trypsin was determined by measuring the activity after carrying out repetitive reactions with trypsin-immobilized chitosan nonwoven fabric. After each use, enzyme-immobilized chitosan nonwoven was removed from the reaction medium and washed twice with 50 mM Tris buffer (pH 8.5) and distilled water. The reusability of immobilized trypsin was evaluated by repeating the above process 15 times [20].

#### 2.2.7. Characterization of Trypsin Immobilization

The surface morphology of the untreated and trypsin-immobilized chitosan nonwoven fabric was observed and compared by using a scanning electron microscope (JSM-7600F, JEOL KOREA LTD., Seoul, Korea). Samples were coated with a thin gold layer, using the fine coater JEOL JFC-1200 and observed. 

## 3. Results and Discussion

### 3.1. Effects of pH, Trypsin Concentration, and Immobilization Time on Trypsin Immobilization

**Figure 3:**
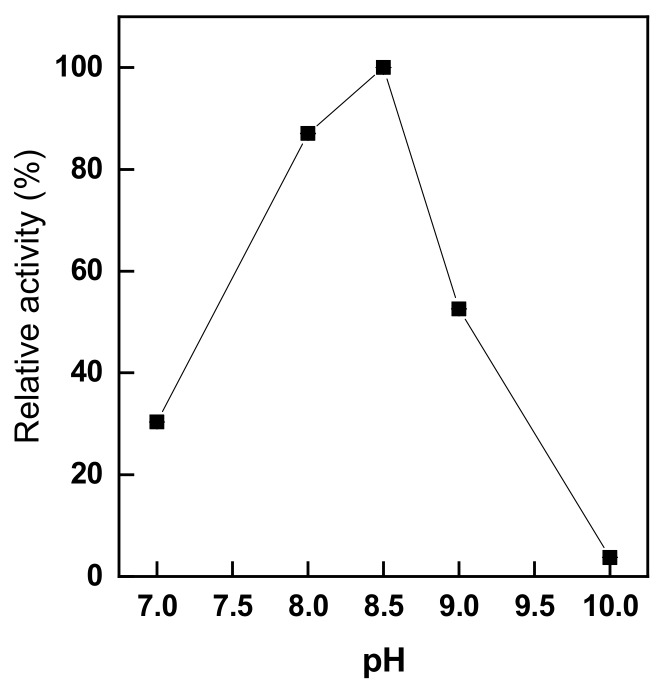
The effects of pH on the immobilization of trypsin onto glutaraldehyde-pre-activated chitosan nonwoven (trypsin immobilization: trypsin conc. 10% (owf), 1 h, 25 °C).

Figure 3 shows the relative activity of immobilized trypsin depending on different immobilization pH values in the range from 7.0 to 10.0 with trypsin concentration of 10% (owf) at 25 °C for 1 h. As shown in Figure 3, the relationship between these two parameters is described by a parabolic shape, and the optimal pH for trypsin immobilization is pH 8.5. This finding replicates the results of another study that showed that the activity of an immobilized enzyme rapidly decreases outside the optimal pH conditions [19]. In particular, we found that the immobilization of trypsin was maximal at pH 8.5, which corresponds with the results of a previous report indicating that the highest amount of the immobilized trypsin was achieved around pH 8.6 [37]. The selection of the optimal pH during immobilization is extremely important because the pH of the immobilization solution affects the structure of the enzyme and the reactivity of the carrier surface [33]. In addition, the activity of trypsin immobilized at pH 10.0 was approximately 3%, which is very low. According to another study, in alkaline conditions, structural changes in trypsin occur and, thus, the activity after immobilization decreases [37]. Therefore, the optimal pH for trypsin immobilization is 8.5, based on the measurements of immobilized-trypsin activity.

**Figure 4:**
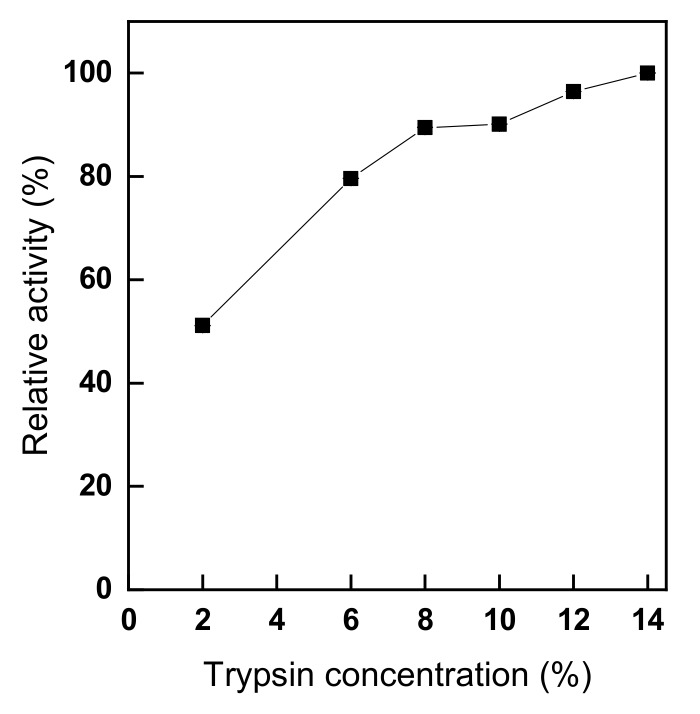
The effect of trypsin concentration on the activity of trypsin immobilized onto glutaraldehyde-pre-activated chitosan nonwoven (trypsin immobilization: pH 8.5, 1 h, 25 °C).

To study the effect of trypsin concentration on trypsin immobilization, trypsin was immobilized on glutaraldehyde-treated chitosan nonwoven using different concentrations of trypsin, ranging from 2% to 14% (owf). As shown in Figure 4, the relative activity of the immobilized trypsin increased linearly as trypsin concentration increased, remained constant between 8% and 10%, and then gradually increased again. The result is similar to those of another study, reporting that the activity of the immobilized enzyme linearly increased up to 2 g/L; in fact, 8% trypsin concentration (owf) corresponds to approximately 1.6 g/L [37]. Moreover, Figure 4 shows that the enzyme activity slowly increased again above the 8% concentration (owf), which can be explained by the onset of trypsin autolysis and enzyme aggregation caused by the high trypsin concentration [2,8,19,33,37]. More specifically, it was determined that the optimal trypsin concentration was 8% (owf), because the slight increase in the activity of immobilized trypsin observed at a concentration above 12% (owf) was due to enzyme aggregation and, further, presented a large margin of error. Not only the enzyme concentration during enzyme immobilization has a significant impact on the immobilization rate and the activity of immobilized enzyme, but also the cost of the enzyme is an important factor that affects the affordability of products.

**Figure 5:**
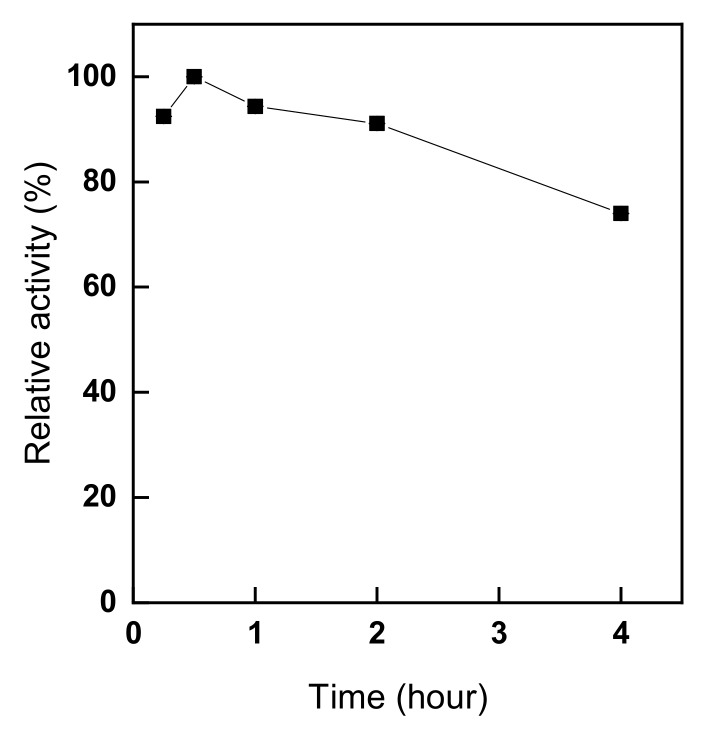
The effects of the immobilization time of trypsin onto glutaraldehyde-pre-activated chitosan nonwoven on the enzyme activity (trypsin immobilization: pH 8.5, trypsin conc. 8% (owf), 25 °C).

Figure 5 shows trypsin activity depending on different immobilization times, from 15 to 240 min at pH of 8.5 with 8% (owf) trypsin at 25 °C. As shown in Figure 5, the activity of immobilized trypsin showed was the highest after 30 min and then decreased with the increase of the immobilization time. Since the amount of aldehyde groups available for immobilization was reduced [2], no further increase of the relative activity of trypsin was observed thereafter. In particular, the activity of immobilized trypsin reached approximately 92% of its maximum value, indicating that immobilization occurred rapidly when immobilizing trypsin on chitosan nonwoven fabric. The above results are similar to those of other studies on the effect of immobilization time on immobilized trypsin activity, where the activity of trypsin after 15 min of immobilization was about 88% of that of the enzyme immobilized for 20 h [37]. Moreover, as in a previous work, after reaching the maximum activity of the immobilized enzyme, the activity decreased, since the active sites of the enzyme were blocked as the immobilization time increased [2]. In other words, as the immobilization time increases, multiple bonds between the enzyme and the support occur, which may change the shape of the enzyme or inactivate its active sites [2,17]. In addition, with increased immobilization time, the increase in Schiff’s bases formed between the enzyme and the support augments the rigidity of the enzyme molecule and reduces the activity of the immobilized enzyme [10]. Therefore, the optimal time of trypsin immobilization based on the trypsin activity measurements was 30 min.

### 3.2. Evaluation of the Stability of Free and Immobilized Trypsin

**Figure 6:**
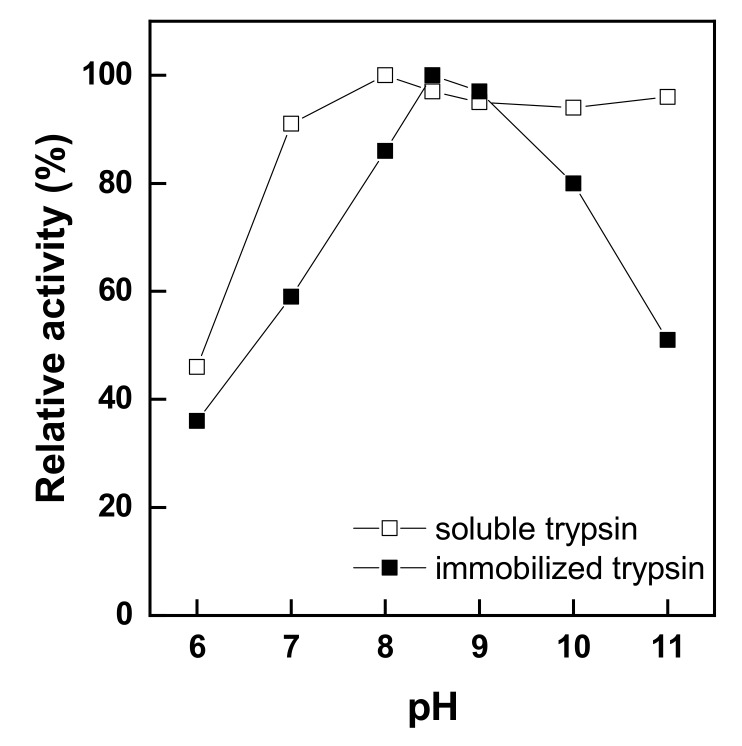
pH stabilities of soluble and immobilized trypsin.

Figure 6 shows the pH stability of free and immobilized trypsin depending on different pH values ranging from 6.0 to 11.0 at 25 °C. The pH stability was evaluated by determining the relative activity of free and immobilized trypsin. As shown in Figure 6, at pH 8.0, the activity was low; however, at pH 8.0, free-trypsin activity showed the maximum value and then slightly decreased and remained stable above pH 8.0. In the case of immobilized trypsin, the highest activity was at pH 8.5; however, the activity declined rapidly at pH values higher than pH 8.5. In other words, immobilized trypsin showed the same activity as free trypsin between pH 8.5 and 9.0 but was stable only within this range, showing a lower stability than free trypsin both below pH 8.0 and above pH 10.0. This implies that immobilized trypsin on glutaraldehyde-pre-activated chitosan nonwoven has a lower stability than free trypsin. The optimal pH for trypsin activity changed with enzyme immobilization, and the stability of the free enzyme was superior at pH values other than the optimal pH for activity [16]. Moreover, the maximum-activity pH moved from pH 8.0 to pH 8.5 after trypsin immobilization because when a fiber-type carrier is used, a higher pH is required due to the decrease in the pH of the active sites of the immobilized enzyme [19,22]. Therefore, the optimal pH was shifted by enzyme immobilization. The pH stability of free trypsin was better than that of immobilized trypsin.

**Figure 7:**
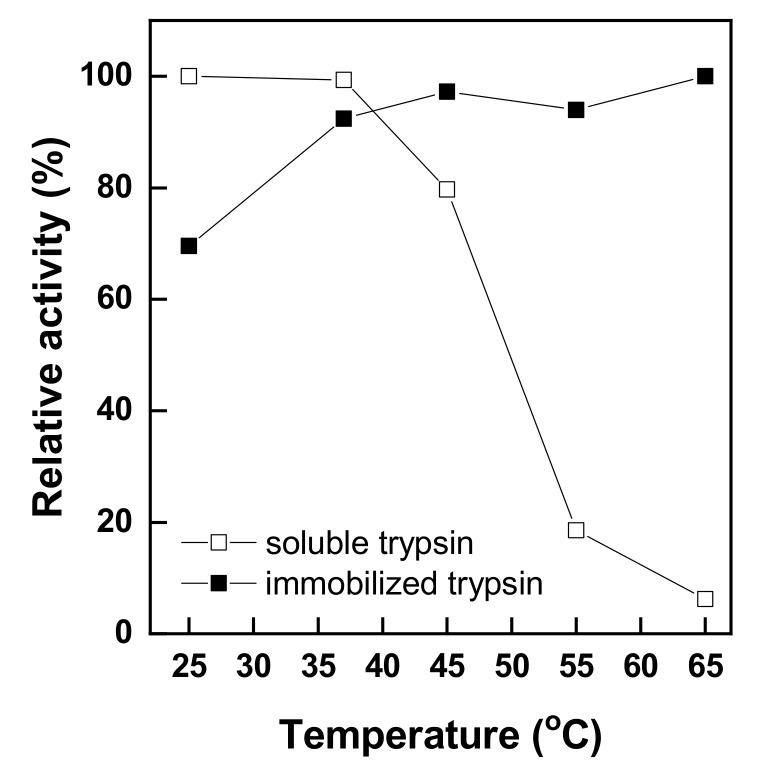
Thermal stabilities of soluble and immobilized trypsin.

Figure 7 shows the thermal stability of free and immobilized trypsin depending on various temperatures from 25 °C to 65 °C. The thermal stability was evaluated on the basis of the relative activity of free and immobilized trypsin. As shown in Figure 7, free trypsin maintained its activity up to 37 °C, but the activity rapidly declined above 37 °C, decreasing to about 6% of the maximum activity. In contrast, the activity of immobilized trypsin increased by about 20% up to 37 °C, compared with its value at 25 °C, somewhat further increased above 37 °C, and remained high at a high temperature of 45–65 °C. The results confirmed that the thermal stability of trypsin increased by immobilizing trypsin on chitosan nonwoven. This was because structural changes and inactivation of trypsin were limited at high temperatures due to the structural rigidity induced by immobilization [38]. Among the immobilization methods, enzyme immobilization by covalent bonding limits denaturation and inactivation by heat observed for free enzymes [3,23]. In addition, the strong bonds between trypsin and chitosan nonwoven protected trypsin from heat-induced structural denaturation and provided high stability against heat [2,10,19,26,38]. Based on these results, immobilized trypsin showed a greater thermal stability than free trypsin; further, immobilization increased the temperature of stable trypsin activity from 37 °C to 45 °C. 

**Figure 8:**
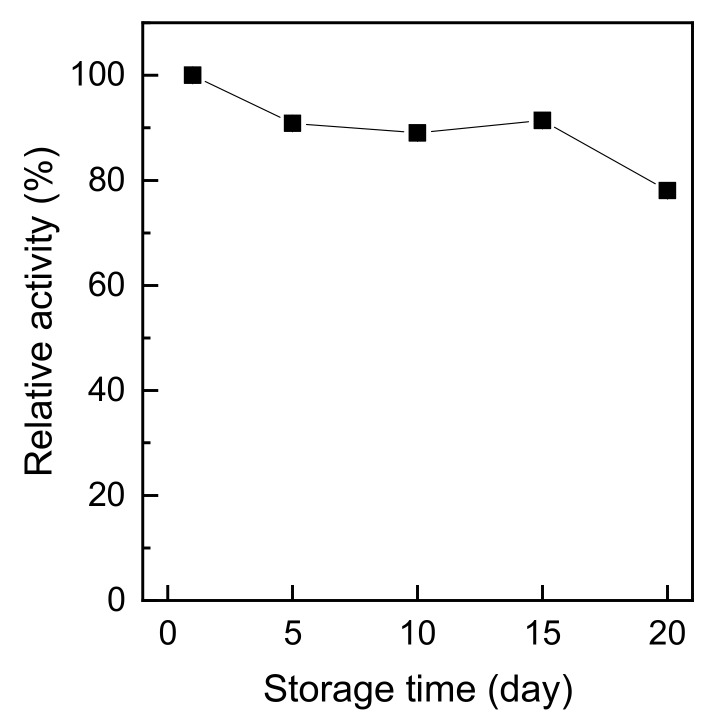
Storage stabilities of immobilized trypsin.

Figure 8 shows the storage stability of immobilized trypsin on chitosan nonwoven. As shown in Figure 8, the relative activity of immobilized trypsin was nearly 90% of the initial activity even after 15 days of storage and decreased by 20% after about 20 days. In general, the activity of the immobilized enzyme decreases with storage time; however, autolysis of trypsin was limited by immobilization, and part of the immobilized trypsin established stronger bonds with the carrier as the storage time increased [31]. The activity of immobilized trypsin declined after 15 days. This is because Schiff’s bases, which formed from the reaction between amino groups and aldehyde groups, are reversible. Despite the strong covalent bond between the enzyme and the carrier, the activity of immobilized trypsin declines rapidly during storage [10]. Based on these results, the storability of the trypsin immobilized on glutaraldehyde-treated chitosan nonwoven by covalent bonds was excellent even after 15 days of storage at 4 °C.

**Figure 9:**
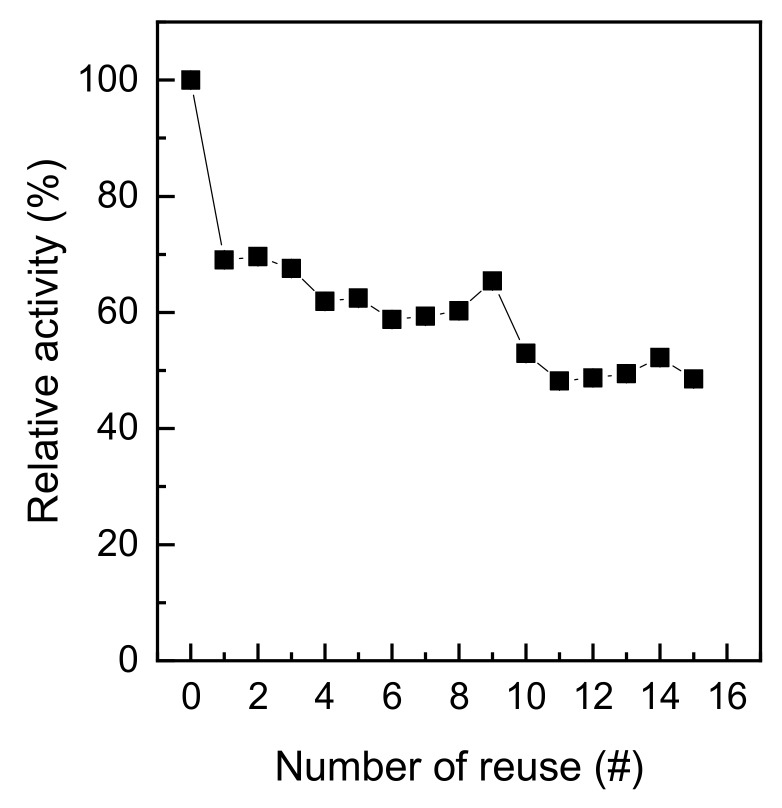
Reusability of immobilized trypsin.

Figure 9 shows the reusability of immobilized trypsin. The reusability of an immobilized enzyme is an important factor that affects the affordability of the enzyme [20]. By using the covalent bonding method, the enzyme and the carrier are more strongly bound than when using physical adsorption or entrapment; hence, the immobilized enzyme could be reused efficiently [10]. As shown in Figure 9, the activity of immobilized trypsin was about 50% of the initial activity even after being used 15 times. This is because the strong bond between glutaraldehyde-pre-activated chitosan nonwoven and trypsin prevented the enzyme loss due to repeated washes [10]. This is similar to the results of another study [20], according to which the activity of immobilized lipase on glutaraldehyde-pre-activated chitosan was 80% of the initial activity even after 10 uses. The results above confirmed the excellent reusability of immobilized trypsin on glutaraldehyde-pre-activated chitosan nonwoven under optimal immobilization conditions. 

According to the results so far, immobilized trypsin had lower pH stability compared to free trypsin; however, it had improved thermal stability, showed 80% of its initial activity after 20 days of storage at 4 °C, and maintained 50% of its initial activity after being used 15 times.

### 3.3. Surface Observation of Trypsin-Immobilized Nonwoven Fabric

**Figure 10:**
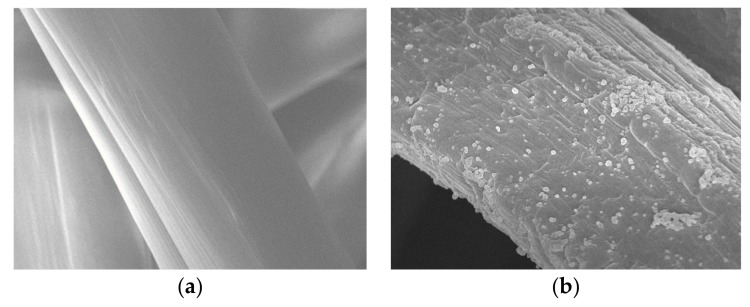
SEM micrographs (×5000) of (**a**) untreated chitosan nonwoven and (**b**) trypsin-immobilized chitosan nonwoven.

Figure 10 shows the results of surface observations of untreated and trypsin-immobilized chitosan nonwoven. In contrast to the smooth surface of untreated chitosan nonwoven fabric shown in Figure 10a, the roughness of chitosan surface increased, presenting round wrinkles after trypsin immobilization, as shown as Figure 10b. The increase in surface roughness was due to trypsin immobilization on the surface of glutaraldehyde-pre-activated chitosan nonwoven [39,40]. In addition, the presence curbed wrinkles on the surface appeared to be due to the recrystallization of trypsin [20,41]. Hence, surface observation confirmed that trypsin was immobilized on chitosan nonwoven.

## 4. Conclusions

In this study, trypsin immobilization on glutaraldehyde-pre-activated chitosan nonwoven was carried out. The optimal immobilization conditions were evaluated considering the pH of the immobilization solution, trypsin concentration, and immobilization time. The pH and thermal stabilities of free and immobilized trypsin were compared. In addition, the storage stability and reusability of immobilized trypsin were evaluated. Moreover, the characteristics of immobilized trypsin were determined by surface observations. The conclusions of this study are as follows: the optimal conditions for trypsin immobilization on glutaraldehyde-treated chitosan nonwoven are pH 8.5, 8% (owf) trypsin concentration, 30 min at 25 °C. The comparison of pH and temperature stabilities of free and immobilized trypsin showed that immobilized trypsin had a lower pH stability than free trypsin, but an excellent thermal stability. In particular, the thermal stability of trypsin to the high temperature of 65 °C was improved by immobilization. In terms of storage stability and reusability, immobilized trypsin showed long storage stability after 20 days of storage at 4 °C and maintained 50% of its initial activity after being used 15 times. The immobilization of trypsin on glutaraldehyde-treated chitosan nonwoven was confirmed through surface observation. This study confirms the development potential of functional textiles using enzyme immobilization. Hence, this study can be utilized in multifunctional textile development research based on enzyme immobilization.

## Figures and Tables

**Table 1:** Characteristics of chitosan nonwoven.

Composition (%)	Thickness (mm)	Weight (g/m^2^)	Manufacturing Method	Type
Chitosan 100	0.38	88.5	Needle punching	Spunlace

**Table 2:** Characteristics of trypsin.

Enzyme	Source	Activity	Form	Manufacturer
Trypsin (EC 3.4.21.4)	*Porcine pancreas*	1000–2000 BAEE units/mg	Lyphophilized powder	Sigma Chemicals Co.
